# Estimating the percentage and number of people with indications for HIV pre-exposure prophylaxis (PrEP) within two key populations, Canada, 2021

**DOI:** 10.17269/s41997-026-01170-5

**Published:** 2026-03-03

**Authors:** Qiuying Yang, Hong-xing Wu, Nathan Lachowsky, Joseph Cox, Ben Klassen, Herak Apelian, Simone Périnet, Janelle Elliott, Anson Williams, Laurence Campeau, Tyrone Curtis, Marcus Wong, Marene Gatali, Nashira Popovic

**Affiliations:** 1 Public Health Agency of Canada, Ottawa, ON Canada; 2 Community-Based Research Centre, Vancouver, BC Canada; 3 University of Victoria, Victoria, BC Canada; 4 McGill University, Montreal, QC Canada

**Keywords:** HIV pre-exposure prophylaxis (PrEP), Indications, HIV prevention, Men who have sex with men (GBMSM), People who inject drugs (PWID), Prophylaxie pré-exposition (PrEP) au VIH, Indications, Prévention du VIH, Hommes ayant des relations sexuelles avec des hommes (HARSAH), Personnes utilisatrices de drogues injectables (UDI)

## Abstract

**Objectives:**

HIV pre-exposure prophylaxis (PrEP) is proven effective for HIV prevention and recommended for individuals at ongoing and high risk of HIV, including gay, bisexual, and other men who have sex with men (GBMSM) and people who inject drugs (PWID). We used existing national survey data to measure PrEP need in both populations and calculate percentages of use among those meeting indications within each group.

**Methods:**

We conducted a secondary data analysis of nationally representative data to estimate the percentage of GBMSM and PWID meeting PrEP indications based on 2017 Canadian guidelines. We applied these percentages to recent population size estimates for both groups to estimate the number of people having PrEP indications in each group. Where available, we also used survey data to determine the percentage of people with indications who were not taking PrEP at the time of the survey.

**Results:**

An estimated 24.1% (95% CI 13.3–39.7%) or 99,300 (95% CI 54,800–163,600) of GBMSM had PrEP indications in Canada as of 2021. The corresponding percentage and number for PWID were 22.3% (20.8–22.8%) and 22,400 (20,900–23,900). Two-thirds of GBMSM participants (Sex Now 2019 and 2021) who had PrEP indications were not taking PrEP at the time of the surveys. None of the participants with PrEP indication in the Tracks survey (2017–2019) among PWID reported PrEP use.

**Conclusion:**

In Canada, the estimated number of people with PrEP indications among GBMSM and PWID far exceeded documented levels of use. Uptake of PrEP was lower among PWID compared to GBMSM. Efforts to improve PrEP access and uptake could help reduce HIV transmission in Canada.

## Résumé

**Objectifs:**

La prophylaxie pré-exposition (PrEP) au VIH est reconnue efficace pour la prévention du VIH et recommandée aux personnes présentant un risque élevé et continu d’infection, notamment les hommes gais, bisexuels et autres hommes ayant des relations sexuelles avec des hommes (HARSAH) ainsi que les personnes utilisatrices de drogues injectables (UDI). Nous avons utilisé des données d’enquêtes nationales existantes pour mesurer les besoins en PrEP dans ces deux populations et calculer les pourcentages d’utilisation parmi les personnes répondant aux indications.

**Méthodes:**

Nous avons réalisé une analyse secondaire de données représentatives à l’échelle nationale afin d’estimer le pourcentage de HARSAH et de personnes UDI répondant aux indications pour la PrEP selon les lignes directrices canadiennes de 2017. Nous avons appliqué ces pourcentages aux estimations récentes de la taille des populations pour estimer le nombre de personnes ayant des indications dans chaque groupe. Lorsque disponible, nous avons également utilisé des données d’enquête pour déterminer la proportion de personnes répondant aux indications qui ne prenaient pas la PrEP au moment de l’enquête.

**Résultats:**

On estime que 24,1% (IC à 95%: 13,3–39,7%) ou 99 300 (IC à 95%: 54 800–163 600) des HARSAH avaient des indications pour la PrEP au Canada en 2021. Le pourcentage et le nombre correspondants pour les personnes UDI étaient de 22,3% (20,8–22,8%) et 22 400 (20 900–23 900). Les deux tiers des participants HARSAH (Sexe au présent 2019 et 2021) ayant des indications pour la PrEP ne la prenaient pas au moment des enquêtes. Aucun des participants ayant des indications dans l’enquête Tracks (2017–2019) auprès des personnes UDI n’a déclaré utiliser la PrEP.

**Conclusion:**

Au Canada, le nombre estimé de personnes ayant des indications pour la PrEP parmi les HARSAH et les personnes UDI dépasse largement les niveaux documentés d’utilisation. L’adoption de la PrEP était plus faible chez les personnes UDI que chez les HARSAH. Des efforts visant à améliorer l’accès et l’utilisation de la PrEP pourraient contribuer à réduire la transmission du VIH au Canada.

## Introduction

In Canada, two of the key populations disproportionately affected by HIV are gay, bisexual, and other men who have sex with men (GBMSM) and people who inject drugs (PWID). In 2023, there were 2434 new HIV diagnoses in Canada, a 35.2% increase from 2022. Of these, 36.0% occurred among GBMSM, 18.2% among PWID, and 4.5% among GBMSM-PWID, accounting for more than half of new diagnoses during that year (Public Health Agency of Canada (PHAC), 2024). However, only an estimated 2.6% of men aged 15 or older have had sex with other men in the past year (Sorge et al., 2023), and 0.28% of the population 15 or older injected drugs in the past year (Williams et al., 2025) in Canada.

Studies have found that HIV pre-exposure prophylaxis (PrEP) is effective in preventing HIV infection in seronegative individuals at substantial risk, including GBMSM and PWID (O Murchu et al., 2022). A recent modelling study showed that reaching 50% PrEP coverage by 2032 among PrEP-eligible GBMSM could avert > 45% of new HIV acquisitions in Atlanta (United States), Montreal (Canada), and the Netherlands (Stansfield et al., 2023). In addition, research from British Columbia, Canada, highlights that optimizing treatment as prevention along with the simultaneous provision of PrEP to high-risk GBMSM could reduce the HIV incidence to as low as 0.4 (0.3–0.6) per 1000 susceptible GBMSM by the end of 2029 (Lima et al., 2021).

Since introducing PrEP in Canada, uptake has increased. In Ontario, following favorable policy changes which included availability of generic tenofovir disoproxil fumarate/emtricitabine and partial public drug coverage (September 2017), including coverage for individuals aged < 25 years (January 2018), PrEP use in Ontario has increased, but remains well below guideline recommendations (Tan et al., 2021). The estimated number of PrEP users was documented to increase by 21-fold, from 460 in 2014 to 9657 in 2018 in eight Canadian provinces (Popovic et al., 2021). However, growth has slowed in recent years, with only a 1.8-fold increase from 13,222 in 2018 to 23,644 in 2021 among nine Canadian provinces (Popovic et al., 2025). Unfortunately, these national data cannot be disaggregated by key populations (i.e., GBMSM, PWID). There is also a lack of studies investigating actual PrEP use among GBMSM and PWID using nationally representative data in Canada.

Knowing the percentage and number of people who need PrEP is important for planning and allocating health resources. As of 2015, the United States (US) Centers for Disease Control and Prevention (CDC) estimated that approximately 24.7% of sexually active GBMSM and 18.5% of PWID in the US were at significant risk for HIV infection and eligible for PrEP (Smith et al., 2015). A Canadian study conducted in Toronto, Vancouver, and Montreal (2017–2019) showed that among 2008 HIV-negative or HIV-unknown sexually active GBMSM, 57.7% met clinical recommendations for PrEP (Cox et al., 2021). A Montreal study from the Hepatitis Cohort (2011–2017) showed 37% PWID met PrEP criteria based on US CDC guidelines (Picard et al., 2020). No study has yet been done on a national scale in Canada.

Using Canadian PrEP guidelines (Tan et al., 2017) and available nationally representative data, we aimed to estimate the percentage and number of GBMSM and PWID in Canada who met PrEP indications. We also examined the percentage of individuals who met PrEP indications but were not taking PrEP among national surveys with available data.

### Methods

This study is a secondary data analysis using existing national survey data. These surveys include the Canadian Community Health Survey (CCHS 2015–2016) conducted by Statistics Canada (Statistics Canada, 2016), two iterations of the Sex Now survey (2019 and 2021), an online survey of gay, bisexual, transgender, and queer men and Two-Spirit and non-binary (GBT2Q) people across Canada (Salway et al., 2021; Lachowsky et al., 2022), and the Tracks biobehavioral survey among PWID across Canada (Tracks survey among PWID, phase 3 (2010–2012) (Tarasuk et al., 2014) and phase 4 (2017–2019) (Tarasuk et al., 2020)). Table 1 provide an overview of these surveys.

**Table 1:** Data sources for estimating the percentage of GBMSM with PrEP indications and of PWID with PrEP indications

Survey	Sample size for estimating PrEP indications	Participating jurisdictions	Recruitment methods
**The percentage of GBMSM with PrEP indications**			
CCHS 2015–2016	575	All provinces and territories	CCHS 2015–2016 employed a multi-stage recruitment process, primarily using area, list, and Random Digit Dialing (RDD) frames to select households for participation. Within these households, individuals were randomly selected, with oversampling of youth and seniors. The survey used a combination of electronic questionnaires and interviewer-led data collection
Sex Now 2019	4481	All provinces and territories	Sampled from general social media platforms (Facebook, Twitter, etc.), geolocating sexual/social networking apps targeted to sexual and gender minority men (Squirt, Scruff, Grindr, etc.), CBRC’s newsletter membership, other community agencies’ newsletter memberships and word-of-mouth. Administered both online and in-person at events across Canada
Sex Now 2021	3114	All provinces and territories	Sampled from general social media platforms (Facebook, Twitter,etc.), geolocating sexual/social networking apps targeted to sexual and gender minority men (Squirt, Scruff, Grindr, etc.), CBRC’s newsletter membership, other community agencies’ newsletter memberships and word-of-mouth Administered online across Canada
**The percentage of PWID with PrEP indications**			
Tracks survey among PWID, phase 3 (2010–2012)	1178	Whitehorse YK, Prince George BC, Edmonton AB, Regina SK, Thunder Bay ON, Sudbury ON, London ON, Toronto ON, Kingston ON, the SurvUDI network (sites in the province of Québec and Ottawa, ON) and Halifax NS	Venue-based sampling, where participants were recruited from settings in which they were likely to gather, most often, but not limited to, needle and syringe distribution programs Sentinel sites were selected based on consultations with provincial/territorial representatives, who considered the epidemiology of HIV, hepatitis C, and drug use and associated harms
Tracks survey among PWID, phase 4 (2017–2019)	1795	Whitehorse YK, Central and Northern Vancouver Island BC, Prince Albert SK, Regina SK, Winnipeg MB, Thunder Bay ON, London ON, Hamilton ON, Ottawa ON and the region of Outaouais QC, Montréal QC, Québec QC, Other urban sites in Quebec, New Brunswick, Newfoundland	Venue-based sampling, where participants were recruited from settings in which they were likely to gather, most often, but not limited to, needle and syringe distribution programs Sentinel sites were selected based on consultations with provincial/territorial representatives, who considered the epidemiology of HIV, hepatitis C, and drug use and associated harms

PrEP indications were based on the 2017 Canadian PrEP guidelines (Tan et al., 2017). However, the variables available in the national surveys did not always align with all criteria outlined in these guidelines. To address these gaps, we also referenced benchmark criteria from US-based CDC study (Smith et al., 2015) and a Montreal-based study (Pichard et al., 2020). Using these sources, we adapted these criteria to fit the data available in Canadian national surveys. Table 2 present the criteria used to identify PrEP indications in these surveys and compare them with the 2017 Canadian PrEP guideline (Tan et al., 2017) and the benchmark studies (Smith et al., 2015, Pichard et al., 2020).

**Table 2:** Criteria for determining PrEP indications among GBMSM and PWID

**Among GBMSM**	
**Source**	**Benchmark criteria**
Canadian PrEP Guideline (Tan et al., 2017)	Condomless anal sex within the last six months AND at least one of the following: Infectious syphilis or rectal bacterial sexually transmitted infection (STI), particularly if diagnosed in the preceding 12 months; Recurrent use of nonoccupational postexposure prophylaxis (nPEP); Ongoing sexual relationship with HIV-positive partner with substantial risk of transmissible HIV; High-incidence risk index (HIRI)-MSM risk score ≥ 11.
US CDC Study (Smith et al., 2015)	Man aged 18–59 years, not known to be HIV-positive and sex with two or more men in the past 12 months. AND at least one of the following: Any reported condomless sex in the past 12 months; STI diagnosis in the past 12 months; HIV status of partners could not be established.
**Surveys**	**Criteria used in this study**
CCHS 2015-2016 (Statistics Canada, 2016)	Men aged 15+, Had sex with two or more men in the past 12 months. AND Did not use a condom during last sexual intercourse with a non-marital, non-cohabiting partner.
Sex Now 2019 (Salway et al., 2021)	Men aged 15+, HIV-negative, Had sex with two or more men in the past 12 months. AND at least one of the following: Did not use a condom during sexual intercourse with a non-marital, non-cohabiting partner in the past 6 months, Diagnosed STI in past 12 months.
Sex Now 2021 (Lachowsky et al., 2022)	Men aged 18+, HIV-negative, Had sex with two or more men in the past 6 months. AND at least one of the following: Did not use a condom during sexual intercourse with a non-marital, non-cohabiting partner in the past 6 months, Diagnosed with or treatment for any STI in past 12 months.
**Among PWID**	
**Source**	**Benchmark criteria**
Canadian PrEP Guideline (Tan et al., 2017)	PrEP may be considered for PWID if they share injection use paraphernalia with a person with a non-negligible risk of HIV infection.
US CDC Study (Smith et al., 2015)	Man or women aged ≥18 years, HIV status could not be determined, and any injection of heroin, methamphetamine, stimulants, or cocaine in the past 12 months. AND at least one of the following Reported injecting with a needle used by someone else before them (other drug preparation equipment not included); Medication-based treatment history could not be assessed; Sex with partner reported to be HIV-positive.
Montreal study (Pichard et al., 2020)	All PWID: those who report injection equipment sharing in the past six months. among heterosexually active males and females: A recently diagnosed bacterial STI; An ongoing sexual relationship with an HIV-positive partner; Infrequent use of condoms during sex with partner(s) of opposite sex who are known to be at substantial risk of HIV infection; or being a behaviourally bisexual male who self-identifies as heterosexual. For sexually active MSM: any condomless anal sex or a recently diagnosed bacterial STI.
**Surveys**	**Criteria used in this study**
Tracks survey among PWID, phase 3 (2010-2012) (Tarasuk et al., 2014)	Individuals aged 15+, who were HIV-negative, who had injected any heroin, methamphetamine, stimulants, or cocaine in past 6 months AND at least one of the following: Reported injecting with a needle used by someone else before them; Sexual partner with HIV-positive or unknown HIV status in the past 6 months; Having casual sex without condom use in the past 6 months.
Tracks survey among PWID, phase 4 (2017-2019) (Tarasuk et al., 2020)	Individuals aged 15+, who were HIV-negative, who had injected any heroin, methamphetamine, stimulants, or cocaine in past 6 months AND at least one of the following: Reported injecting with a needle used by someone else before them; Sexual partner with HIV-positive or unknown HIV status in the past 6 months; Having casual sex without condom use in the past 6 months.

Overall pooled estimates for the percentage of individuals having PrEP indications with inverse-variance weights were obtained from a random-effects model. These percentages were estimated using the metaprop command in R (Suvarnapathaki, 2021). We then applied these pooled percentages to the most recent Canadian population size estimates for GBMSM (Sorge et al., 2023) and PWID (Williams et al., 2025) to estimate the number of individuals with PrEP indications in each group.

We also used data from online surveys of GBT2Q men (Salway et al., 2021; Lachowsky et al., 2022) and the Tracks survey among PWID Phase 4 (–) (Tarasuk et al., 2020) to examine the percentages of GBMSM and PWID with PrEP indications who were not taking PrEP at the time of the survey. Analyses were performed using SAS EG 7.1 and R version 4.2.1. Absolute numbers were rounded to three significant digits.

## Results

**Fig. 1:**
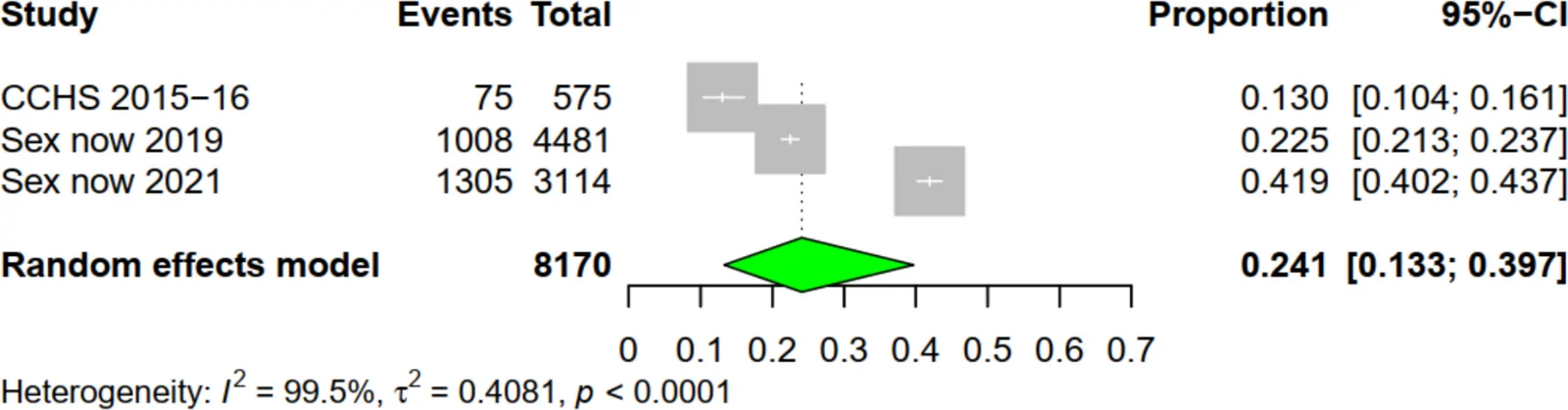
Estimated percentage (95% CI) of GBMSM with PrEP indications by survey

Across surveys among GBMSM in Canada, the percentage of GBMSM meeting PrEP indications ranged from 13.0% (95% confidence intervals (CI) 10.4–16.1%) to 41.9% (95% CI 40.2–43.7%). The pooled estimate using a random-effects model was 24.1% (95% CI 13.3–39.7%). Figure 1 presents individual survey estimates, the random-effects model estimate, and corresponding confidence intervals. Due to substantial heterogeneity and temporal variability in survey criteria, interpretation should focus on the 95% confidence intervals as a range of plausible values rather than relying on the point estimate as a single stable construct.

We found that the majority of participants (77.9% in Sex Now 2019 and 84.7% in Sex Now 2021) were eligible for PrEP, based on response to the question related to not using a condom during sexual intercourse with a non-marital, non-cohabiting partner in the past 6 months. Approximately one-third of participants (33.3% in Sex Now 2019 and 31.9% in Sex Now 2021) had been diagnosed with or received treatment for any STI in the past 12 months. Some participants met both criteria.

**Fig. 2:**
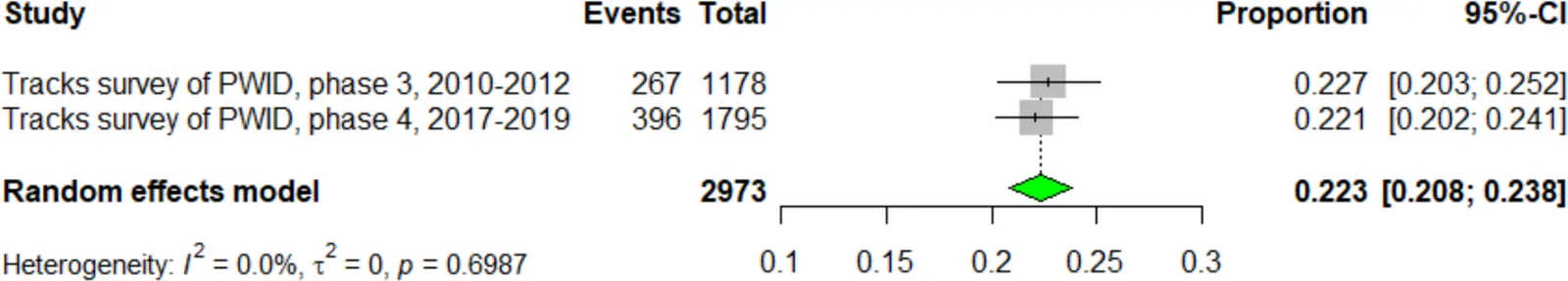
Estimated percentage (95% CI) of PWID with PrEP indications by survey

The pooled percentage of PWID having PrEP indications was estimated using a random-effects model, since surveys were conducted in different sites and years. The estimated percentage of PWID with PrEP indications was 22.3% (95% CI 20.8–23.8%). Figure 2 illustrates the percentage and 95% CI of PWID with PrEP indications for each survey.

Approximately half of participants (64.0% in the Tracks survey among PWID, phase 3 (2010–2012) and 51.2% in the Tracks survey among PWID, phase 4 (2017–2019)) were eligible for PrEP use because they reported having injected with a used needle within the previous 6 months, whereas approximately half of participants (49.8% in the Tracks survey among PWID, phase 3 (2010–2012) and 58.3% in the Tracks survey among PWID, phase 4 (2017–2019)) were eligible for PrEP use because they reported having engaged in casual sex without a condom within the previous 6 months. Some participants met both criteria.

There were an estimated 412,186 GBMSM representing 2.6% of the male population aged 15 years and older (Sorge et al., 2023), and 100,300 PWID representing 0.28% of the total population aged 15 years and older in Canada (William A et al., 2025). Therefore, we estimated that 99,300 GBMSM (95% CI 54,800–163,600) and 22,400 PWID (95% CI 20,900–23,900) had PrEP indications in Canada as of 2021 (Table 3).

**Table 3:** Size of GBMSM and PWID as well as percentage and number of GBMSM and PWID with PrEP indications

	Estimated population size	Estimated % with PrEP indications	Estimated number with PrEP indications
GBMSM	412,186	24.1% (95% CI 13.3–39.7%)	99,300 (95% CI 54,800–163,600)
PWID	100,300	22.3% (95% CI 20.8–22.8%)	22,400 (95% CI 20,900–23,900)

Regarding real-world use of PrEP, 68.8% (Sex Now 2019) and 66.9% (Sex Now 2021) of those with PrEP indications were not taking PrEP at the time of the survey. The Tracks survey among PWID, phase 4 (2017–2019) documented that no participants with PrEP indications were taking PrEP during the survey period.

## Discussion

The surveys included in the meta-analysis suggest an increasing trend in the percentage of PrEP indications over time among GBMSM. This trend likely reflects a combination of factors, including rising STI rates during this period (Nguyen et al., 2018; von Schreeb et al., 2024) and declining condom use reported in behavioral datasets (Newcomb et al., 2018). However, the questionnaires used in the source surveys differed slightly, and there is no conclusive evidence of increased condomless anal sex associated with PrEP use (Daroya et al., 2024). These limitations prevent us from determining whether the observed trend is due to actual secular changes or to differing criteria used across surveys.

Based on our findings, an estimated 99,300 (95% CI 54,800–163,600) GBMSM and 22,400 (95% CI 20,900–23,900) PWID met PrEP indications in 2021. However, according to available data from nine Canadian provinces (representing 88% of the Canadian population), only an estimated 23,644 people were using PrEP in 2021 (Popovic et al., 2025). The estimated number of people with PrEP indications among GBMSM and PWID far exceeded documented levels of use. This suggests that, according to 2017 guideline recommendations (Tan et al., 2017), many individuals who could benefit from PrEP were not currently using it in these two key populations. Access to PrEP expanded significantly in eight Canadian provinces from 2014 to 2018, with a 21-fold increase (Popovic et al., 2021). The upward trend in PrEP use continued in nine Canadian provinces from 2018 to 2021, but at a slower pace, with a 1.8-fold increase (Popovic et al., 2025). Updated Canadian PrEP guidelines were released recently (Tan et al., 2025). These revised guidelines expand the eligibility criteria and encourage more healthcare providers to prescribe PrEP, potentially improving uptake among those at higher risk of HIV infection.

Our findings show that among GBMSM aged 15 years and over who reported sexual activity in the past year, approximately 24.1% (95% CI 13.3–39.7%) had PrEP indications. In a similar study from the US CDC, this percentage was comparable at 25% for 2015 (Smith et al., 2015). However, a Canadian study conducted from 2017 to 2019 in Toronto, Vancouver, and Montreal found that among 2008 HIV-negative or HIV-unknown sexually active GBMSM, 57.7% met clinical recommendations for PrEP (Cox et al., 2021). Compared with this study, the surveys we used had less information on PrEP use indications recommended by Canadian guidelines, which may have underestimated the percentage with PrEP indications. However, the study conducted by Cox et al. used a respondent-driven sampling method to recruit participants, which may have recruited similar high-risk participants and thus may have overestimated the percentage with PrEP indications.

We found that 22.3% (95% CI 20.8–22.8%) of individuals aged 15 years and over who reported injecting drugs in the past 6 months had PrEP indications. This is slightly higher than the 18% reported in a US CDC study for 2015 (Smith et al., 2015), but lower than the 37% observed in a Montreal study conducted from 2011 to 2017 (Picard et al., 2020). In the Montreal study, 25.7% of PWID met the criteria of sharing injection equipment in the last 6 months while the corresponding numbers were 14.5% in the Tracks survey among PWID, phase 3 (2010–2012), and 11.1% in the Tracks survey among PWID, phase 4 (2017–2019). The Montreal study population was drawn from the Hepatitis Cohort, which may have recruited more high-risk participants, potentially explaining the higher percentage meeting PrEP indications (Picard et al., 2020).

The Sex Now surveys found that despite the impact of COVID-19 on health services (PHAC, 2022), PrEP use among GBMSM who met indications was stable over the past 2 years. Specifically, 31.2% of eligible participants reported current PrEP use in 2019 and 33.1% in 2021. These percentages are higher than those reported in previous studies. For example, an online survey conducted between 2017 and 2018 in Canada (Colyer et al., 2021) found that 24.6% of participants were currently using PrEP. Two in three people with PrEP indications among GBMSM in the Sex Now surveys were not taking PrEP at the time of the survey; among PWID, on the other hand, uptake was even lower, with no individuals reporting PrEP use at the time of the survey in the Tracks survey among PWID, phase 4 (2017–2019). There is a lack of recent studies measuring PrEP use among PWID in Canada. However, data from the National HIV Behavioral Surveillance (NHBS) from San Francisco, US, showed that PrEP uptake in 2022 was 1.2% among HIV-negative PWID representing negligible change since it was previously measured in NHBS in 2018 (Suprasert et al., 2022).

Numerous barriers to PrEP acceptability and use have been identified in the literature. These include HIV-related stigma (Golub & Gamarel, 2017), self-perception of low HIV risk (Cox et al., 2021; Kesler et al., 2016), misconceptions about who PrEP is for (Thomann et al., 2018), health system barriers (Etowa et al., 2022), cost (Pico-Espinosa et al., 2023), adherence challenges with oral PrEP (Hosek et al., 2016), and concerns about potential side effects (Kesler et al., 2016). Addressing these barriers may increase PrEP uptake among individuals with indications for use in Canada.

Lima VD et al. (2021) assert that HIV elimination is possible in British Columbia by a combined strategy of Treatment as Prevention and PrEP use. Therefore, expanding PrEP use through tailored approaches in Canada could reduce new HIV infections in the future. Nurse-led PrEP and patient-initiated continuing medical education for primary care physicians are feasible strategies to increase PrEP uptake in Canada as well (Charest et al., 2021). In addition, ensuring adherence to PrEP will be important. Research has demonstrated that harmful alcohol use and moderate-/high-risk cocaine use independently predicted nonadherence to PrEP use among GBMSM in Toronto (Shuper et al., 2020). These findings highlight the need to integrate alcohol- and substance-focused initiatives into PrEP care for GBMSM (Shuper et al., 2020). Finally, long-acting, injectable PrEP provides another potential means to expand coverage and possibly address adherence for some key populations (Miller et al., 2023).

There are several limitations to our study. First, not all indications in the Canadian PrEP guideline could be directly matched to the variables collected in the surveys analyzed in this study. Second, the Sex Now, Tracks survey among PWID, and CCHS data may be subject to measurement error, recall bias, and reporting bias due to self-reporting, as described in their respective methodologies. In addition, some surveys may have inherent limitations related to non-probabilistic sampling (Salway et al., 2021). Third, the most recent available Tracks survey among PWID was conducted during 2017–2019, which coincided with policy changes in some Canadian provinces regarding access to and cost of PrEP. For example, British Columbia implemented a publicly funded PrEP program in January 2018, offering free access to eligible residents through the BC Centre for Excellence in HIV/AIDS. Ontario introduced generic tenofovir disoproxil fumarate/emtricitabine with partial public drug coverage in September 2017, expanding coverage to individuals under 25 years in January 2018. Alberta followed with a no-cost PrEP program for individuals at high risk of HIV starting October 1, 2018. These policy shifts may have influenced PrEP uptake and access during the survey period, potentially affecting the interpretation of results. Fourth, recent versions of the CCHS surveys (2017–2023) did not collect sexual behavior information, necessitating the use of older data from the 2015–2016 cycle, which may not reflect recent patterns. Furthermore, the CCHS sample was subject to a greater degree of social desirability bias due to its interviewer-administered management. This may have led to fewer reports of anal sex with men in the CCHS survey than in the Sex Now survey (Sorge et al., 2023). However, due to its promotion on sexual/social networking apps, the Sex Now sample might recruit more sexually active GBT2Q individuals. We used pooled estimates to estimate the overall percentages that may offset over- or under-estimation. Despite these limitations, our results may slightly underestimate the PrEP indications in these two key populations; however, we believe our results fall within the plausible range.

Effective PrEP policies, including inclusive eligibility criteria, sustainable funding mechanisms, and accessible delivery models, are crucial to ensuring that PrEP benefits those who need it. PrEP should be integrated with other HIV prevention strategies, such as testing and treatment in order to maximize its public health impact. Well-designed and inclusive PrEP policies can increase PrEP uptake and adherence, ultimately helping to reduce HIV transmission across Canada.

## Conclusions

In Canada, the estimated number of people with PrEP indications among GBMSM and PWID populations currently exceeds the annual estimated number of people prescribed PrEP. These findings suggest that more can be done to increase the use of PrEP in these key populations. Uptake of PrEP was substantially lower among PWID compared to GBMSM, underscoring the need for targeted strategies to improve access and engagement within this group. Increasing the awareness of PrEP among individuals at greater risk and among health care providers through health promotion initiatives, as well as improving the access to and availability of PrEP, in combination with other effective HIV prevention methods, can contribute to reducing the number of new HIV infections that occur each year in Canada.

## Contributions to knowledge

What does this study add to existing knowledge?

- To the best of our knowledge, this study provides the first national estimates of the percentages and numbers of gay, bisexual, and other men who have sex with men (GBMSM) and people who inject drugs (PWID) in Canada who meet indications for PrEP based on Canadian guidelines.
- It identifies an increasing trend in the percentage of GBMSM meeting PrEP indications over time.
- The study quantifies the gap between PrEP indications and actual use; approximately two-thirds of GBMSM who had PrEP indications were not taking PrEP at the time of the surveys, and none of the PWID with indications was using PrEP during the surveys.

What are the key implications for public health interventions, practices, or policy?

- The estimated number of people with PrEP indications among GBMSM and PWID far exceeded documented levels of use in Canada. These findings underscore the need to scale up public health programs that increase awareness, access, and uptake of PrEP among these populations to reduce HIV transmission.
- Updated Canadian PrEP guidelines, which expand the eligibility criteria and encourage prescribing, were released recently. Training and support for healthcare providers will be essential to ensure these guidelines are implemented effectively.
- Ongoing nationwide biobehavioral surveys by PHAC will provide an opportunity to update the estimated percentage of PrEP indications based on revised guidelines in the future.

## Data Availability

Not applicable.
